# Frequent and Recent Non-fatal Strangulation/Choking During Sex and Its Association With fMRI Activation During Working Memory Tasks

**DOI:** 10.3389/fnbeh.2022.881678

**Published:** 2022-06-02

**Authors:** Megan E. Huibregtse, Isabella L. Alexander, Lillian M. Klemsz, Tsung-chieh Fu, J. Dennis Fortenberry, Debby Herbenick, Keisuke Kawata

**Affiliations:** ^1^Department of Kinesiology, School of Public Health, Indiana University, Bloomington, IN, United States; ^2^Center for Sexual Health Promotion, School of Public Health, Indiana University, Bloomington, IN, United States; ^3^Department of Applied Health Science, School of Public Health, Indiana University, Bloomington, IN, United States; ^4^Department of Pediatrics, School of Medicine, Indiana University, Indianapolis, IN, United States; ^5^Program in Neuroscience, College of Arts and Sciences, Indiana University, Bloomington, IN, United States

**Keywords:** functional neuroimaging (fMRI), verbal working memory, visual working memory, sex behavior, non-fatal strangulation/choking

## Abstract

Being strangled, or “choked,” by a sexual partner has emerged as a prevalent, often wanted and consensual sexual behavior among adolescent and young adult women, yet the neurological consequences of repeated exposure to this behavior are unknown. The objective of the study was to examine the association between a history of repeated, recent choking/strangling episodes during sex and fMRI activation during working memory tasks in young adult women. This case-control study involved young adult women (18–30 years old) at a large, public university, and consisted of two study groups: a choking group consisting of participants who were recently and frequently choked/strangled during sex by a partner (≥4 times in the past 30 days) and a choking-naïve (control) group who had never been choked/strangled during sex. Participants completed two variations of the N-back (0-back, 1-back, and 2-back) working memory task during functional magnetic resonance imaging (fMRI): verbal and visual N-back tasks. Data from 20 participants per group were available for analysis. Between-group differences for accuracy and reaction time were not significant for either variation of the N-back task. Significant differences in fMRI activation patterns were detected between the choking and the choking-naïve groups for the three contrasts of interest (1-back > 0-back, 2-back > 0-back, and 2-back > 1-back). The choking group exhibited increased activation in multiple clusters relative to the choking-naïve group for the contrasts between the 1-back and 2-back conditions compared to the 0-back conditions (e.g., superior frontal gyrus, corpus callosum). However, the choking-naïve group exhibited increased activation relative to the choking group in several clusters for the 2-back > 1 back contrast (e.g., splenium, middle frontal gyrus). These data indicate that recent, frequent exposure to partnered sexual strangulation is associated with different neural activation patterns during verbal and visual working memory tasks compared to controls, suggesting that being choked/strangled during sex may modify the allocation of neural resources at increasing levels of cognitive load. Further investigation into the neurologic effects of this sexual behavior is warranted, given the prevalence of sexual choking among adolescent and young adult women.

## Introduction

Being strangled, or “choked” as it is colloquially termed, has emerged worldwide as a popular behavior in partnered sexual activities, as it may increase pleasure and lead to euphoric feelings when oxygen supply is returned to the brain and is part of a general increase in rough sex behaviors (Sun et al., 2017; Herbenick et al., 2020, 2021a; Wright et al., 2021). Strangulation/choking as a partnered sex behavior is disproportionately experienced by women. For instance, in a recent undergraduate probability survey study, nearly one-third of undergraduate women reported being choked by a partner during their most recent sexual event that included oral, vaginal, or anal sex, compared to only 8% of men (Herbenick et al., 2021b). Further, 58% of undergraduate women reported a lifetime history of at least one instance of being choked during partnered sexual activities, with 34% reporting more than five lifetime choking experiences (Herbenick et al., 2021a). In contrast, only 6% of undergraduate men reported being choked more than five times (Herbenick et al., 2021a). While depictions of choking in pornography can lead viewers to believe that choking a sexual partner is pleasurable and safe (Wright et al., 2021), choking is commonly considered to be a rough sex behavior that may increase the risk of injury in a sexual encounter (Herbenick et al., 2019). Being choked by a sexual partner was often described as a scary experience in a population-based probability survey of Americans between 14 and 60 years old (Herbenick et al., 2019), and strangulation is a common component of intimate partner violence (IPV) against women (McQuown et al., 2016; Pritchard et al., 2017). Strangulation, even with non-fatal intentions, carries inherent health risks. For example, strangulation was identified as the most common cause of death in Bondage, Discipline, Dominance, Submission, Sadism, and Masochism (BDSM)-related fatalities in a literature review of case reports from 1986 to 2020 (Schori et al., 2022).

Strangulation compresses or completely blocks blood vessels in the neck (the carotid arteries and the jugular vein) and/or blocks the airway, leading to decreased cerebral blood flow (cerebral ischemia) and oxygen availability (cerebral hypoxia), both of which can induce brain damage with only minimal force required (De Boos, 2019; Bichard et al., 2021). Following a period of reduced blood flow, the return of blood flow and oxygen to the deprived tissues can result in damage from ischemia-reperfusion injury, characterized by oxidative stress and inflammation (Kalogeris et al., 2012). Survivors of non-fatal strangulation in the context of IPV and sexual assault report headaches, dysphasia, ptosis, post-traumatic stress disorder and other emotional reactions to trauma, and cognitive difficulties, such as memory problems, and confusion (Bichard et al., 2021). Additionally, being frequently choked by a sexual partner has been linked to worse mental health in a recent probability survey of undergraduate students, as women who had been choked more than five times in the past month were more likely to endorse feeling sad, lonely, anxious, and depressed compared to women without a history of being choked (Herbenick et al., 2021a). While the effects of choking as a partnered sex behavior may be distinct from the effects of non-fatal strangulation in IPV and sexual assault, they may share some characteristics. Despite the increased attention on this behavior within the field of sexual health and IPV research, the neurological and neurobehavioral consequences of repetitive exposure to being non-fatally strangled by a sexual partner in adolescent and young adult women have not yet been examined.

Therefore, we conducted a pilot case-control study to evaluate the impact of frequent exposure to sexual strangulation on working memory in young adult women recruited from a large public university into two groups: a choking group who reported four or more instances of being choked in the past 30 days by a sexual partner and a choking-naïve control group without any history of being choked by a partner during a sexual event. Participants completed verbal and visual N-back working memory tasks during functional magnetic resonance imaging (fMRI) to examine the potential association between frequent and recent exposure to being strangled by a sexual partner and alterations in fMRI activation during working memory tasks. First, we hypothesized that the recent, frequent choking group and the choking-naïve group would not exhibit overt differences in behavioral performance, in terms of accuracy and reaction time, as even individuals with a history of mild traumatic brain injury (mTBI), with commonly reported symptoms of memory problems and difficulty concentrating (Katz et al., 2015), often do not differ in terms of N-back working memory task performance from healthy controls (McAllister et al., 1999; Terry et al., 2015; Shah-Basak et al., 2018). Second, we hypothesized that the choking group would exhibit altered blood oxygen level dependent (BOLD) signal patterns during both tasks relative to the choking-naïve group, as strangulation may induce cerebral hypoxia and/or ischemia followed by cerebral reperfusion injury.

## Materials and Methods

### Experimental Design and Recruitment

This case-control study was conducted from February to June 2021. The Indiana University Institutional Review Board approved the study and written informed consent was obtained from all participants. Research participants were recruited *via* two mechanisms: (1) respondents in a separate university-wide survey study could indicate that they were interested in being contacted about a follow-up study on sexual behaviors and were asked to provide their email address; and (2) additional participants were recruited using the university online classifieds section.

**Figure 1:**
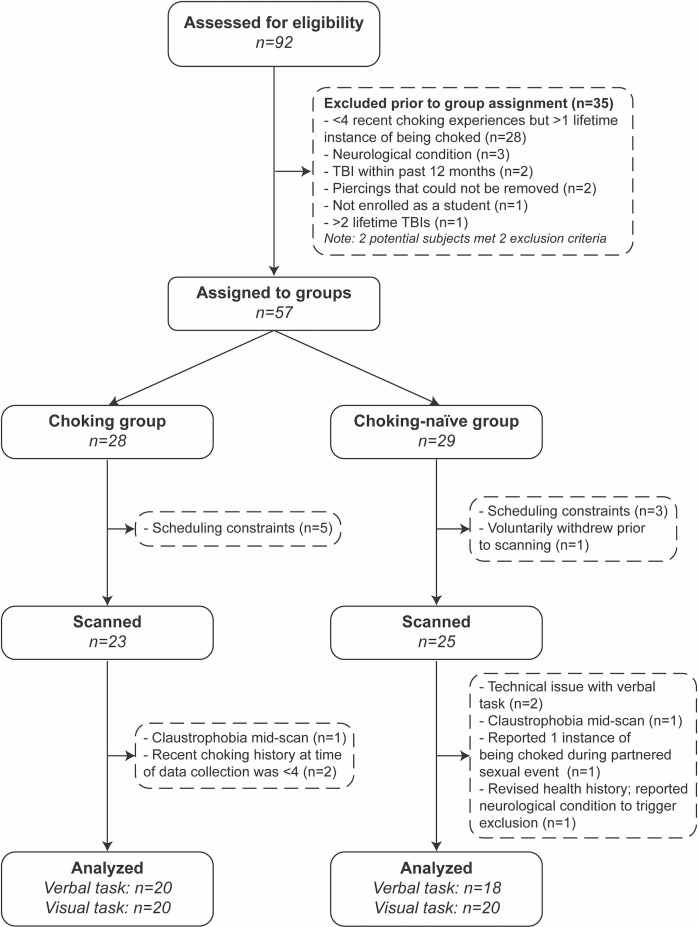
Study flow chart.

Participants completed a screening questionnaire to determine eligibility and group assignment. For inclusion, participants were required to be female, be between 18 and 30 years old, and be enrolled at Indiana University—Bloomington. General exclusion criteria included being pregnant, reporting a traumatic brain injury within the past year, reporting more than two lifetime traumatic brain injuries, having any MRI contraindications (e.g., recent tattoos that were incompletely healed, severe claustrophobia, metal implants unsafe for 3T MRI scanning), neurological conditions (e.g., epilepsy or recent history of seizures, neurodegenerative disease, aneurysm, brain tumor, spinal cord injury). Additional exclusion criteria were used to determine group assignment. For the choking group, individuals were excluded if they reported fewer than four instances of being choked by a partner during sexual events in the past 30 days. For the choking-naïve group, individuals were excluded if they reported having ever been choked/strangled by a partner during a sexual event. Eligible participants were then assigned to groups and scheduled for a data collection session (see Figure 1).

### Neuroimaging Parameters

All participants were scanned on a Siemens 3T Prisma scanner, equipped with a 64-channel head/neck coil. Anatomical images (T1 weighted) were acquired using 3D MPRAGE pulse sequence with the following parameters: TR/TE = 2,400/2.3 ms, TI = 1,060 ms, flip angle = 8, matrix = 320 × 320, bandwidth = 210 Hz/pixel, iPAT = 2, which resulted in 0.8 mm isotropic resolution.Task-based whole brain functional images were acquired with multi-slice single-shot echo-planar sequences (FOV = 216 mm, TR/TE = 800/30 ms, flip angle = 52°, matrix = 90 × 90, resolution = 2.4 mm isotropic, multiband acceleration factor = 6). The start of the tasks was triggered by the scanner following the dummy scans.MRI data quality was checked at multiple stages throughout the data collection and analysis processes. First, the subject’s head motion was monitored in real-time using Framewise Integrated Real-time MRI Monitoring software (FIRMM, NOUS Imaging, St. Louis, MO) and excessive motion would have prompted a second run of the affected scan. No participants had to repeat scans for excessive motion. A trained, experienced MRI operator visually inspected all scans for noticeable artifacts—none were observed. Data quality following preprocessing was visually checked by the first author for each participant prior to proceeding to the first- and second-level analyses (see descriptions below).

### N-Back Working Memory Tasks

Participants completed two tasks: (1) verbal N-back working memory task; and (2) visual N-back working memory task. For the verbal N-back task (0-, 1-, and 2-back), the stimuli were capital letters: B, C, D, F, G, H, J, K, L, M, N, P, Q, R, S, T, V, W, X, and Z. For each condition, the participant was shown the target (0-back), the first letter in the series (1-back), or the first two letters in the series (2-back) for 2,000 ms per letter. Then a series of letters were displayed for 2,000 ms each, and the participant was asked to respond by pushing the button under their left index finger if the letter on the screen matched the target letter, matched 1-back, or matched 2-back (depending on the condition) or by pushing the button under their right index finger if the letter did not match. The trial type (match or not a match) was randomly selected, with a total of four matches and six non-matches per condition. Conditions were presented in ascending order (0-back, 1-back, and 2-back) and repeated for a total of four repetitions. Conditions were separated by 10 s rest intervals. The duration for the entire verbal N-back task was 392 s.

The visual N-back working memory task (0-, 1-, and 2-back) followed the same task design structure as the verbal N-back task, except the stimuli were 12 simple abstract line drawings. For the first two repetitions of the three conditions, the stimuli were presented in one orientation. For the second two repetitions of the three conditions, task difficulty increased as some of the stimuli appeared in multiple orientations, and the participants were instructed to determine if it was a match based on both shape and orientation. The duration for the entire visual N-back task was 400 s, 8 s longer than the verbal task due to the presentation of instructions to start matching based on shape and orientation for the second half of the task. Participants were given instructions and shown demo screens for both tasks prior to entering the scanner room, and instructions were given again immediately prior to each task.

### Questionnaires

Following the MRI scan, participants completed a series of pen-and-paper questionnaires. Participants provided demographic information, a general health history, and information on sexual health and behavior. Participants were asked to indicate if they had been sexually active in the past year (“*Have you been sexually active in the past year (12 months)?*”), if they had ever been choked/strangled (“*Have you ever been choked by a partner (e.g., they pressed or squeezed your neck with their hands, arm or an object) during a sexual event/experience (e.g., romantic kissing, sexual touching, oral sex, vaginal or anal sex, sex toy use, etc.)?*”), and, if they indicated that they had been choked during a sexual event, approximately how many times they had been choked in the past year (12 months), past 60 days, and the past 30 days.

Participants also completed scales assessing symptoms of depression [Patient Health Questionnaire—Depression Module (PHQ-9)] and anxiety [Generalized Anxiety Disorder 7-Item Scale (GAD-7)], in addition to the Alcohol Use Disorders Identification Test (AUDIT). The PHQ-9 assesses depression-related symptoms: each of the nine items describes one symptom corresponding to one of the nine Diagnostic and Statistical Manual of Mental Disorders (fourth edition; DSM-IV) diagnostic criteria for depression (Spitzer et al., 1999; Kroenke and Spitzer, 2002). The GAD-7 assesses symptoms of anxiety disorders in terms of presence and severity (Spitzer et al., 2006; Kroenke et al., 2010). The AUDIT is a 10-item screening tool developed by the World Health Organization (WHO) to assess alcohol consumption, drinking behaviors, and alcohol-related problems (Bohn et al., 1995; Bush et al., 1998).

### Statistical Analysis

#### Demographic and Mental Health Variables

Differences between the choking group and the choking-naïve group were assessed for the demographic and mental health variables. After checking for normality with the Shapiro-Wilk tests, Mann-Whitney *U* tests were used to compare the groups for the continuous variables (e.g., age, PHQ-9 score, GAD-7 score, AUDIT score). Categorical variables (e.g., student status, race, sexually active) were compared between groups using chi-square tests. All tests were two-tailed, and statistical significance was set *a priori* at *p* < 0.05. Analysis of demographic and mental health variables was performed using R (version 4.1.2).

#### Task Performance Variables

To evaluate the first hypothesis, accuracy, in terms of percent of correct responses, and mean reaction time were calculated for each task condition and each participant, after excluding any responses with reaction times less than 200 ms. These responses were omitted from behavioral analysis due to the extreme likelihood that they did not represent deliberate responses, as these response times were faster than biologically plausible reaction times with the minimum visual, information, and motor production processing required to respond (Nuri et al., 2013; Jain et al., 2015). Accuracy and reaction time were analyzed separately for each task using mixed-effects linear regression models to determine the main effects of task condition and group, in addition to the group-by-condition interaction effects. Any significant main effects were further examined using Bonferroni’s correction for multiple comparisons testing. All tests were two-tailed, and statistical significance was set *a priori* at *p* < 0.05. Task performance analyses were performed using Prism 9 (version 9.0.1; GraphPad, San Diego, CA, USA).

#### fMRI Preprocessing and Analysis

All anatomical and functional images were preprocessed and analyzed using SPM12^1^. Preprocessing steps included: (1) realignment; (2) co-registration of the structural and functional images; (3) normalization of the co-registered images to Montreal Neurological Institute (MNI) space; and (4) smoothing with a Gaussian kernel with a full width at half maximum (FWHM) of 8 mm. Slice timing correction was not performed due to the short TR (800 ms).

The fMRI data were first analyzed at the participant level (first-level) by fitting general linear models (GLM) for each participant. A boxcar function was used to model the onsets and durations of the rest periods, 0-back conditions, 1-back conditions, and 2-back conditions, convolved with a canonical hemodynamic response function. Contrast images were generated for the three contrasts of interest: 1-back > 0-back, 2-back > 0-back, and 2-back > 0-back. These three contrasts were chosen to examine group differences in activation patterns for working memory “maintenance” (1-back > 0-back), “maintenance plus manipulation” (2-back > 0-back), and “manipulation only” (2-back > 1-back), as described by Ragland et al. (2002). Six motion parameters (x, y, and z translation and rotation) were estimated during preprocessing and then included as covariates in the first level analysis. The individual contrast images for all participants in the two groups were then entered into the second level (i.e., group level) random-effects models. Between-group whole-brain differences for each of the contrasts of interest were examined using two-sample *t*-tests with non-parametric permutation testing (5,000 permutations) for each contrast of interest. The overlapping regions were identified using the MNI coordinates from the non-parametric permutation analysis results and the Harvard-Oxford and Johns Hopkins University atlases in MRIcron^2^.

## Results

### Demographic and Mental Health Variables and Recent Choking/Strangulation Frequency

A total of 92 individuals gave written consent, and 57 participants were assigned to groups after eligibility screening (see Figure 1). Twenty-three and 25 participants were scanned for the choking and choking-naïve groups, respectively. The final sample sizes, less the participants who experienced claustrophobia and ended the scan early, reported exclusion criteria at the time of data collection or were unable to complete a task due to technical difficulties, where *N* = 20 for both tasks for the choking group and *N* = 18 and *N* = 20 for the choking-naïve group for the verbal and visual tasks, respectively. Demographic and health history variables were compared for the full groups (see Table 1). The recent, frequent choking group was younger, comprised proportionally more undergraduates, and had more racial-ethnic diversity than the choking-naïve group. More participants in the choking group indicated that they had been sexually active in the past 12 months, compared to the choking-naïve group, and the choking group had slightly higher AUDIT scores.

**Table 1:** Demographic characteristics.

**Variable**	**Choking group**	**Choking-naïve group**	***p* value**
N	20	20	-
Sex, female, *n* (%)	20	20	-
Age, years, median (IQR)	21 (20 – 22)	23 (21 – 26)	0.014
Student status, *n* (%)			0.009
Undergraduate student	17 (85%)	8 (40%)	
Graduate student	3 (15%)	12 (60%)	
Race, *n* (%)^a^			0.010
American Indian/Alaskan Native	1 (5%)	0 (0%)	
Asian	4 (20%)	3 (15%)	
Black/African American	5 (25%)	0 (0%)	
White	14 (70%)	17 (85%)	
No. of experiences being choked by a sexual partner, median (IQR)			-
Past 30 days	7.0 (5.0 – 12.8)	0	
Past 60 days	15.5 (10.0 – 26.3)	0	
Past 12 months	42.5 (20.0 – 60.0)	0	
Sexually active in past 12 months, *n* (%)	20 (100%)	13 (65%)	0.013
PHQ-9, median (IQR)	5.00 (2.75 – 8.25)	4.00 (0.00 – 6.50)	0.37
GAD-7, median (IQR)	6.50 (2.75 – 10.00)	4.50 (0.00 – 6.25)	0.11
AUDIT, median (IQR)	4.50 (3.00 – 7.00)	3.00 (1.00 – 4.25)	0.026

*Note: PHQ-9, Patient Health Questionnaire—9; assesses depressive symptoms; GAD-7, General Anxiety Disorder—7; assess anxiety symptoms; AUDIT, Alcohol Use Disorders Identification Tool; screens for unhealthy alcohol use. IQR, interquartile range; SD, standard deviation. ^a^Several individuals in the choking group indicated that they identified as more than one race/ethnicity, so the percentages add up to more than 100%. For the chi-square test used to compare race between groups, these individuals were coded as multiracial*.

### Behavioral Performance Did Not Differ Between Groups Across the Three Conditions of the N-Back Working Memory Tasks

**Figure 2:**
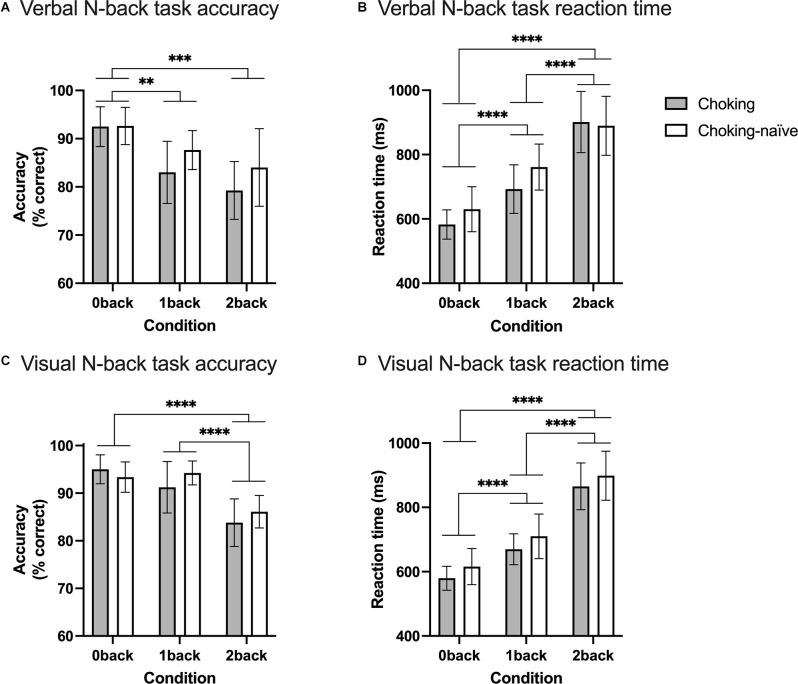
Working memory task performance, in terms of accuracy and reaction time. Accuracy **(A)** and reaction time **(B)** for the verbal N-back working memory task and accuracy **(C)** and reaction time **(D)** for the visual N-back working memory task are shown using bars and error bars to depict means and 95% confidence intervals, respectively. The significance of depicted pairwise comparisons is represented with asterisks as follows: *p < 0.05, **p < 0.01, ***p < 0.001, ****p < 0.0001.

The choking group and choking-naïve group did not differ in terms of response accuracy across the three conditions for either N-back task, as reflected by nonsignificant group × condition interactions, in line with our first hypothesis (verbal N-back task: *F*_(2, 72)_ = 0.658, *p* = 0.521; visual N-back task: *F*_(2, 76)_ = 1.291, *p* = 0.281). Accuracy decreased as N increased, as supported by a significant main effect of condition (verbal N-back task: *F*_(1.7, 61.7)_ = 11.7, *p* = 0.001; visual N-back task: *F*_(1.8, 67.1)_ = 20.4, *p* < 0.001; see Figures 2A,C). Multiple comparisons testing revealed significant decreases in accuracy on the verbal N-back task from 0-back to 1-back (adjusted *p* = 0.0068) and 0-back to 2-back (adjusted *p* = 0.0006). The difference in accuracy on the verbal N-back task did not differ between the 1-back and 2-back conditions. For the visual N-back task, there was no difference in accuracy between the 0-back and 1-back conditions, but the decreases in accuracy from 0-back to 2-back (adjusted *p* < 0.0001) and from 1-back to 2-back (adjusted *p* < 0.0001) were significant.

Likewise, the two groups had similar reaction times across the three conditions for both N-back tasks, as supported by nonsignificant group × condition interaction effects (verbal N-back task: *F*_(2, 72)_ = 1.384, *p* = 0.257; visual N-back task: *F*_(2, 76)_ = 0.0178, *p* = 0.982), in support of our first hypothesis. As N increased, reaction time slowed (or increased) for both tasks, with significant main effects of condition (verbal N-back task: *F*_(1.8, 65.3)_ = 67.6, *p* < 0.0001; visual N-back task: *F*_(1.8, 68.3)_ = 121.1, *p* < 0.0001; see Figures 2B,D). All pairwise comparisons between conditions for both tasks were significant (adjusted *p* < 0.0001 for all comparisons).

### fMRI Activation Patterns Differed Between Groups During the Verbal N-Back Task

**Figure 3:**
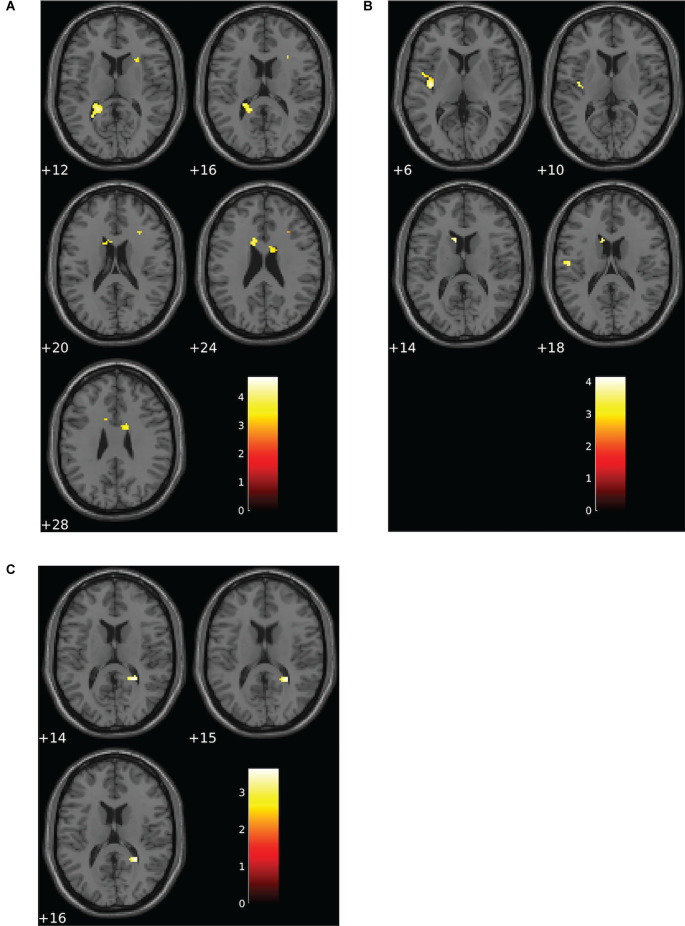
Group differences for patterns of BOLD signal for the verbal N-back task. **(A)** The choking group exhibited increased activation compared to the choking-naïve group for the contrast between the 1-back and 0-back conditions. **(B)** For the contrast between the 2-back and 0-back conditions, the choking group exhibited increased activation compared to the choking-naïve group. **(C)** For the contrast between the 2-back and 1-back conditions, the choking-naïve group had increased activation in one cluster relative to the choking group. Statistical non-parametric maps were thresholded at *p* = 0.001 (uncorrected) and *k* ≥ 20.

Altered patterns of activation were observed during the verbal task in the choking group relative to the choking-naïve group, partially confirming our second hypothesis. During the verbal N-back task, increased activation was detected in multiple clusters in the choking group compared to the choking-naïve group for the 1-back > 0-back and 2-back > 0-back contrasts (see Table 2). Notably, large clusters were observed bilaterally in the corpus callosum and in the L posterior thalamic radiation for the 1-back > 0-back contrast and in the L insula and L caudate for the 2-back > 0-back contrast (see Figures 3A,B). No significant activation clusters were observed in the choking-naïve group compared to the choking group for these contrasts.

**Table 2:** Activation details for significant activation clusters for group comparisons on verbal N-back working memory task.

**Contrast**	***k*_E_**	**T**	**Overlapping region(s)**	**Peak MNI coordinates**			***p* value**
				***x***	***y***	***z***	
**1-back > 0-back**							
choking>choking-naïve	166	4.70	L posterior thalamic radiation; edge of L occipital lateral ventricle	−28	−50	10	<0.001
	50	4.13	L corpus callosum	−10	10	22	<0.001
	31	3.85	R external capsule	26	18	12	0.001
	54	3.65	R corpus callosum	14	4	28	<0.001
	24	2.74	R caudate; edge of R frontal lateral ventricle	8	8	6	<0.001
2-back > 0-back							
choking > choking-naïve	24	4.14	L caudate; edge of L frontal lateral ventricle	−8	14	14	<0.001
	77	4.06	L posterior insula	−38	−12	6	<0.001
	22	3.62	L postcentral gyrus	−50	−14	18	0.001
2-back > 1-back							
choking-naïve > choking	26	3.64	R splenium; edge of R lateral ventricle atrium	26	−46	16	<0.001

*Note: *k_E_*, cluster extent (in voxels); *T*, non-parametric t-statistic; MNI, Montreal Neurological Institute; SFG, superior frontal gyrus. Statistical non-parametric maps were thresholded at *p* = 0.001 (uncorrected) and k ≥ 20*.

Subtle yet significant between-group differences were detected for the 2-back > 1-back contrast for the verbal N-back task, such that increased activation was detected in one small cluster (R splenium) in the choking-naïve group compared to the choking group (see Table 2, Figure 3C).

### fMRI Activation Patterns Differed Between Groups During the Visual N-Back Task

**Figure 4:**
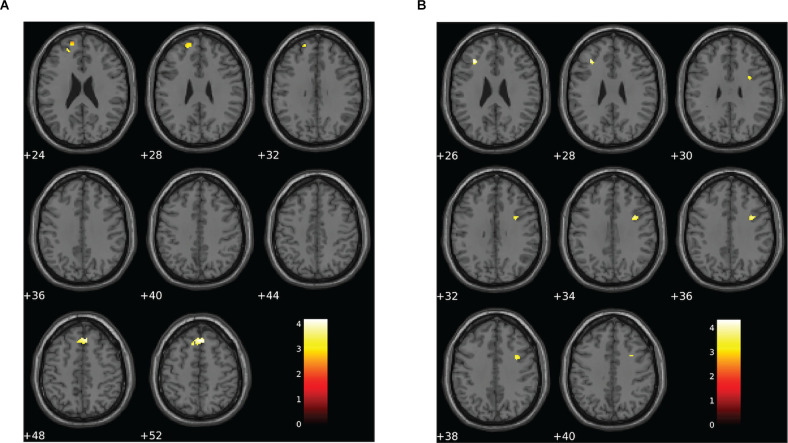
Group differences for patterns of BOLD signal for the visual N-back task. **(A)** The choking group exhibited two clusters of increased BOLD signal for the contrast between the 1-back and 0-back conditions compared to the choking-naïve group. **(B)** The opposite pattern was observed for the contrast between the 2-back and 1-back conditions, such that the choking-naïve group exhibited increased activation in two clusters relative to the choking group. There were no significant differences between groups for the patterns of BOLD signal for the contrast between the 2-back and 0-back conditions for the visual N-back task. Statistical non-parametric maps were thresholded at *p* = 0.001 (uncorrected) and *k* ≥ 20.

Altered patterns of activation were also observed during the visual N-back task in the choking group relative to the choking-naïve group, providing additional evidence in support of our second hypothesis. Like the verbal N-back task, increased activation was detected in the choking group relative to the choking-naïve group in several clusters for the contrast between the 1-back and 0-back conditions in the visual N-back task (see Table 3, Figure 4A). Namely, we observed two clusters of increased bilateral activation in the superior frontal gyrus and a small cluster in the R middle frontal gyrus. There were no significant clusters of increased activation in the choking-naïve group compared to the choking group for the 1-back > 0-back contrast, and no significant between-group clusters were detected for the 2-back > 0-back contrast.

**Table 3:** Activation details for significant activation clusters for group comparisons on visual N-back working memory task.

**Contrast**	***k*_E_**	**T**	**Overlapping regions**	**Peak MNI coordinates**			***p* value**
				***x***	***y***	***z***	
1-back – 0-back							
choking > choking-naïve	135	4.19	R SFG	4	30	52	0.001
	78	3.43	L SFG (PFC)	−22	46	24	0.001
2-back – 1-back							
choking-naïve > choking	24	4.33	L MFG	−32	30	26	<0.001
	57	3.88	R MFG	36	6	34	0.001

*Note: *k_E_*, cluster extent (in voxels); *T*, non-parametric t-statistic; MNI, Montreal Neurological Institute; SFG, superior frontal gyrus; PFC, prefrontal cortex; MFG, middle frontal gyrus; IFG, inferior frontal gyrus. Statistical non-parametric maps were thresholded at *p* = 0.001 (uncorrected) and k ≥ 20*.

For the 2-back > 1-back contrast, several significant clusters of increased activation were detected in the bilateral middle frontal gyrus and the right inferior frontal gyrus in the choking-naïve group relative to the choking group (see Table 3, Figure 4B). No significant clusters were detected in the choking group relative to the choking-naïve group for the 2-back > 1-back contrast.

## Discussion

This is, to our knowledge, the first study to examine the effects of frequent, recent exposure to being choked/strangled during partnered sexual activities on neurological function as assessed by the BOLD signal during N-back working memory tasks. The results supported our first hypothesis that the two groups would not differ in behavioral performance. While an increase in N resulted in decreased accuracy and slower reaction times, the choking and the choking-naïve groups did not significantly differ in terms of accuracy or reaction time for both the verbal and visual N-back working memory tasks. Additionally, we observed altered blood oxygen level dependent (BOLD) signal patterns during both tasks in the choking group relative to the choking-naïve group, supporting our second hypothesis. The choking group generally exhibited increased activation compared to the choking-naïve group for the 1-back > 0-back and 2-back > 0-back contrasts for both tasks, and the choking-naïve group exhibited increased activation relative to the choking group for the contrasts between the 2-back and 1-back conditions for both tasks.

The overall pattern of group differences observed in the present study suggests that additional neural resources are allocated for demands of working memory maintenance in the choking group, while the choking-naïve group exhibits more neural activation for target object/letter manipulation throughout the task. Specifically, we observed increased activation in clusters in the bilateral superior frontal gyri and in the right middle frontal gyrus in the choking group compared to the choking-naïve group for the contrast between the 1-back and 0-back conditions of the visual N-back working memory task. These regions are critical for working memory, often in a load-dependent manner (Rypma et al., 1999; Song and Jiang, 2006). Interestingly, we observed significant differences in BOLD signal between groups for the 2-back > 1-back contrast for both tasks, such that the choking-naïve group exhibited increased activation compared to the choking group in several clusters, albeit different clusters for each variant of the N-back task. The clusters with increased activation in the verbal working memory task were in the splenium, while the clusters with increased activation in the visual working memory task were concentrated in the middle frontal gyrus. It should be reiterated that all participants in this study were current students enrolled in undergraduate or graduate courses and were dedicating time and effort to academic study, supporting the expected finding that the two groups performed similarly in terms of accuracy and response reaction time. Further, fMRI task performance often does not differ between individuals with mTBI and healthy controls despite significant differences in activation patterns (McAllister et al., 1999; Shah-Basak et al., 2018). While the cluster sizes of differences in activation are somewhat small (ranging from 22 to 166 voxels), we want to highlight our use of non-parametric permutation testing for the two-sample *t*-tests, which has been shown to be more conservative and less erroneous than the parametric statistical analysis (Eklund et al., 2016). Further, as we used a relatively conservative *p*-value threshold (*p* < 0.001) and the clusters were not very large and did not span multiple anatomical regions/structures, cluster-extent based inferences for activation in the regions identified using the peak activation MNI coordinates are spatially specific (Woo et al., 2014). Altogether these results suggest that even with similar levels of task performance between the choking and choking-naïve groups, being choked at least four times during sex in the past month is associated with different patterns of the BOLD signal during working memory tasks.

As awareness of choking/strangulation as a partnered sexual behavior has entered mainstream conversations, questions concerning the safety and long-term psychological and neurologic consequences of being choked/strangled in this context have been raised (Herbenick et al., 2021e). Recent and frequent exposure to being choked has been linked to worse mental health in a recent probability survey of undergraduate students. Undergraduate women with a history of being choked more than five times during sex within the past 30 days were 2.19 times as likely to endorse experiencing overwhelming anxiety, 2.16 times more likely to report feeling very sad, 1.59 times more likely to report being very lonely, and 1.77 times more likely to feel “so depressed that it was difficult to function” than women who had never been choked (Herbenick et al., 2021a). However, it should be noted that IPV, IPV-related strangulation, sexual assault, and adverse childhood experiences were not examined or controlled for by Herbenick et al. (2021a), and the authors acknowledged that the examination of these variables would enrich future investigations of choking as a sexual behavior. Within the context of IPV, a history of being non-fatally strangled has been associated with worse cognitive functioning (Valera et al., 2022). Women with histories of strangulation-related alterations in consciousness (AIC) performed significantly worse on measures of both long-term and working memory compared to women who had experienced IPV but had never experienced strangulation-related AIC (Valera et al., 2022). There are three important distinctions to be made between the study reported by Valera et al. (2022) and the present work. First, Valera et al. (2022) utilized validated and sensitive neuropsychological tests, including the California Verbal Learning Test (Delis et al., 1987) and the Digit Span of the Wechsler Adult Intelligence Scale-Revised (Wechsler, 1981) to examine memory function, while the present study used reaction time and response accuracy during the fMRI tasks to compare behavioral performance between the choking and the choking-naïve groups. As mentioned above, fMRI task performance, in terms of accuracy and reaction time, is often not impaired in patients with acute mTBI (McAllister et al., 1999; Shah-Basak et al., 2018) and therefore may not be as sensitive as validated neuropsychological tests. Second, the two studies examine different types of choking/strangulation exposure. Strangulation-related alterations in consciousness were defined by Valera et al. (2022) as events that resulted in the individual experiencing symptoms of hypoxic or ischemic brain injury (e.g., dizziness, feeling stunned or disoriented, seeing stars or spots, losing consciousness, or blacking out, or posttraumatic amnesia) while or after being choked by an intimate partner in the absence of blunt force trauma to the head. While these incidents of psychological and physical trauma in the form of IPV may have resulted in chronic alterations in functional and structural neural integrity, the comparison to an IPV-exposed group without a history of strangulation-related AIC supports the conclusion that it is perhaps the history of strangulation-related AIC that is associated with worse cognitive functioning in this group (Valera et al., 2022). For the present study, we were interested in the frequency of incidents during which a sexual partner had choked them during sex, which we described as “pressed or squeezed your neck with their hands, arm, or an object”—without any kind of provision about physical sensations or choking intensity. On a related note, choking/strangulation exposure for group assignment purposes in the present study was restricted to the past month, while the time since the most recent strangulation in Valera et al. (2022) ranged from 1 week to 21 years before the data collection sessions. Lastly, Valera et al. (2022) recruited participants (mean age of 32 years) primarily from help-seeking populations (shelters, relationship support programs, substance abuse support programs), as the focus of that investigation was IPV-related strangulation. As our objective was to examine the effects of strangulation/choking as a partnered sexual behavior in young adult women, we recruited women from the general student body at a large, public university, and the median ages of the choking and the choking-naïve groups were 21 and 23 years, respectively.

The present study reveals an association between a history of being choked recently and frequently during sex and patterns of fMRI activations during verbal and visual N-back working memory tasks. However, a longitudinal investigation would permit causal inference between partaking in this sexual behavior and altered patterns of neural activation during cognitive tasks. Additionally, a longitudinal investigation allows researchers to delineate the role of dose and time-interval of choking/strangulation on neurophysiological alteration. Potential explanations for this type of relationship include repetitive cerebral hypoxic-ischemic and reperfusion injury, functional and structural remodeling of brain networks as a consequence of repetitive participation in this sexual behavior or even a combination of these two mechanisms. Being choked/strangled during sex can block the airway and/or occlude major blood vessels carrying oxygen, glucose, and other nutrients to the brain. The brain is particularly vulnerable to hypoxia and ischemia due to its high oxygen and glucose consumption requirements, low levels of antioxidant activities, and structural components particularly susceptible to oxidative damage (Kalogeris et al., 2012). Without a sufficient supply of oxygen, cells must rely on anerobic metabolism and adenosine triphosphate (ATP) is depleted. Once blood flow is re-established and the tissue is reperfused, reactive oxygen species (ROS) are generated, damaging proteins and lipids, and pro-inflammatory mediators are produced, triggering cell death mechanisms (Busl and Greer, 2010), leading to changes in functional and structural integrity over time as demonstrated by a rodent model of global cerebral ischemia and reperfusion injury (Wang et al., 2020). Moreover, the experience of being choked repetitively may alter functional and structural brain networks. Being choked, even if done for reasons related to increasing pleasure and arousal, is sometimes described as frightening and even terrifying (Herbenick et al., 2019, 2021d). Experience-dependent changes in neural plasticity and function have been well documented in both rodent models and humans. Stress, in the form of psychosocial stress (Liston et al., 2009), exogenous glucocorticoid administration (Lupien et al., 2007), and experience of traumatic events (Jeong et al., 2019), can disrupt functional networks and impair working memory. Adolescence and young adulthood constitute a critical development period during which the brain is particularly sensitive to the effects of experience (Berardi et al., 2015). Thus, being choked frequently during sex could result in changes in neural structure and function, and this effect could be compounded when this behavior is experienced during late adolescence and early adulthood.

### Limitations

The results of this pilot case-control study should be interpreted within the context of several limitations. First, the group assignment relied on self-reported choking/strangulation history, which raises two concerns. The self-reported choking/strangulation history within the choking group was both subject to recall bias (Bradburn et al., 1987) and spanned a wide range of frequencies. Thus, while all participants assigned to the choking group reported at least four instances of being choked/strangled in the past 30 days, the choking/strangulation exposure within the choking group was relatively heterogenous. Second, we did not ask or evaluate if they had experienced an AIC (lost consciousness or blacked out) during any of these recent incidents of choking. Losing consciousness would suggest that sufficient pressure was placed on the carotid arteries, the jugular vein, and/or the airway (Bichard et al., 2021). Thus choking-related AIC could serve as an important indicator for adverse neurological consequences. Further, if some of the participants had experienced AIC during recent choking incidents while others did not, additional heterogeneity in terms of potential choking effects may be present within the choking group. Additionally, we did not determine the time since the most recent choking incident. Therefore, we cannot speak to the possible influences of acute exposure to this behavior. Third, due to participant recruitment constraints related to the COVID-19 pandemic, we were unable to match participants in the choking-naïve group to participants in the choking group. Ideally, the choking-naïve group should have been at minimum matched in terms of age and sexual activity status to participants in the choking group. On a related note, recruitment and data collection began prior to the expansion of COVID-19 vaccine access to this age group in our state. Therefore, it is possible that some individuals began to alter their behaviors (e.g., initiation of new sexual relationships, more frequent in-person interactions) as they got vaccinated. Fourth, we did not collect data on which or if any of the instances of being choked/strangled by a sexual partner were consensual, nor did we examine prior stressful and/or traumatic life experiences (e.g., IPV, adverse childhood experiences, sexual assault). If the instances of choking occurred in frightening and/or nonconsensual circumstances, as suggested by Herbenick et al. (2021c), representing traumatic and stressful experiences, it is plausible that this component could contribute to the altered patterns of fMRI activation during the working memory tasks. Acute and chronic effects of stress have been linked to widespread changes in activation and connectivity in the brain (van Oort et al., 2017; Kunimatsu et al., 2020). That said, the majority of sexual choking events have been described as consensual, wanted, and often initiated by women (Herbenick et al., 2021b,d,e). Fifth, we did not assess tendencies to participate in risky sex or sensation-seeking behaviors, which may be contributing factors to engaging in sexual choking. Indeed, sensation-seeking has been linked to engaging in risky sexual behaviors (Donohew et al., 2000; Turchik et al., 2010) and increased resting-state functional connectivity between the medial orbitofrontal cortex and the anterior cingulate cortex (Wan et al., 2020). Further, Hansen et al. (2018) reported that risky sexual behavior was associated with increased BOLD signal in the right dorsolateral prefrontal cortex during response inhibition (incongruent > congruent contrast; Stroop task) in adolescents aged 14–18 years old. Sensation-seeking should be assessed in future studies examining sexual choking and neural function. Lastly, we restricted participant eligibility to female students, reducing the applicability of these preliminary findings to men, but this decision was supported by two reasons. Previous literature suggests being choked/strangled, both in cases of IPV and as a partnered sexual behavior, is highly gendered, with significantly more women being choked/strangled as compared to men (McClane et al., 2001; Herbenick et al., 2021e). Additionally, sex differences have been reported in working memory behavioral performance (Voyer et al., 2017, 2021) and fMRI activation during working memory tasks (Bell et al., 2006; Zilles et al., 2016).

### Conclusion

We aimed to examine the association between a history of being choked/strangled during sex and working memory function and task performance. Overall, young women with a history of being choked during sex exhibited different patterns of fMRI activation during verbal and visual working memory tasks compared to a group of peers with no history of being choked during sex. Given the prevalence of this behavior and its preliminary associations with altered working memory function and worse mental health, future research should aim to address the limitations of the present work, examine additional cognitive processes, such as emotional processing and response inhibition, and employ a longitudinal design to investigate a potentially causal relationship between being choked and negative neurologic and mental health outcomes.

## Data Availability Statement

The raw data supporting the conclusions of this article will be made available by the authors, without undue reservation.

## Ethics Statement

The study involving human participants was reviewed and approved by Indiana University Institutional Review Board. The participants provided their written informed consent to participate in this study.

## Author Contributions

MH, T-cF, JF, DH, and KK contributed to the conception and design of the study. MH, IA, and LK collected the data. MH performed the statistical analysis and wrote the first draft of the manuscript. All authors contributed to the article and approved the submitted version.

## Conflict of Interest

The authors declare that the research was conducted in the absence of any commercial or financial relationships that could be construed as a potential conflict of interest.

## Publisher’s Note

All claims expressed in this article are solely those of the authors and do not necessarily represent those of their affiliated organizations, or those of the publisher, the editors and the reviewers. Any product that may be evaluated in this article, or claim that may be made by its manufacturer, is not guaranteed or endorsed by the publisher.

## Footnotes

^1^ https://www.fil.ion.ucl.ac.uk/spm/software/spm12/

^2^ https://www.nitrc.org/projects/mricron
